# Blood-conserving and therapeutic efficacy of intravenous tranexamic acid at different time points after primary total knee arthroplasty with tourniquet application: a randomised controlled trial

**DOI:** 10.1186/s12891-023-07036-y

**Published:** 2023-11-17

**Authors:** Mingyou Wang, Yuping Lan, Hongping Wang, Chunyu Chen, Zhu Mei, Qifeng Tao

**Affiliations:** Department of Orthopaedics, Panzhihua Central Hospital, 34# Yikang road, Panzhihua, Sichuan 617000 People’s Republic of China

**Keywords:** Tranexamic acid, Tourniquet, Total knee arthroplasty, Blood loss, Ecchymosis and swelling, Application and study

## Abstract

**Background:**

The use of a tourniquet in combination with tranexamic acid (TXA) not only ensures clear vision, reduces intraoperative blood loss and shortens operative time but also improves cement-bone inter-digitation in total knee arthroplasty (TKA). However, there is no proof whether the blood flow blocking effect of tourniquet affects the antifibrinolytic effect of TXA, and the optimal timing of TXA administration is still unclear. Therefore, this study aims to investigate the effect of the first dose of TXA administered intravenously before tourniquet compression and release in TKA on perioperative blood loss and therapeutic efficacy in patients.

**Methods:**

In this double-blind trial, 90 patients undergoing primary TKA were randomised into 2 groups: Group A, patients received intravenous TXA 10 min before tourniquet compression (20 mg/kg) and 3, 6 and 24 h later (10 mg/kg), and Group B, patients were treated the same as those in Group A but received intravenous TXA before tourniquet release. The primary outcomes were changes in blood loss, haemoglobin and haematocrit. Secondary outcomes included operation and tourniquet times, blood transfusion rate, subcutaneous petechiae and circumferential changes in the operated limb, visual analogue scale (VAS) score, hospital for special surgery (HSS) score, length of stay (LOS) postoperatively, complications and patient satisfaction.

**Results:**

No statistically significant difference was found between the 2 groups with regard to age, sex, weight, body mass index (BMI), Kellgren-Lawrence class, preoperative blood volume, preoperative laboratory values, operation and tourniquet times, transfusion rate, knee circumference, preoperative HSS, or VAS score (*P*:n.s.). There was no significant difference in intraoperative blood loss (IBL) (52.7 ml vs. 63.4 ml, *P* = 0.07), hidden blood loss (HBL) (91.4 ml vs. 119.9, *P* = 0.4) or total blood loss (TBL) (144.1 ml vs. 183.3 ml, *P* = 0.72) between Groups A and B. Haemoglobin, haematocrit and red blood cell count (RBC) dropped to a low point on postoperative day 3 and then rebounded, returning to normal levels on day 21, and the trend of change between the 2 groups was not statistically significant (*P*:n.s.). There was no significant difference in subcutaneous ecchymosis incidence, knee swelling rate, HSS score, VAS score, LOS postoperatively, complication rate or patient satisfaction (*P*:n.s.).

**Conclusion:**

TXA was administered intravenously prior to tourniquet compression could effectively reduce blood loss in patients who had undergone total knee arthroplasty. However, there was no significant difference in knee swelling rate, subcutaneous bruising and petechiae incidence, knee function, complication rate or satisfaction between patients who TXA was given intravenously before tourniquet compression and release in primary TKA.

## Introduction

Total knee arthroplasty (TKA) has become a routine procedure to reduce pain and improve the quality of life of patients with osteoarthritis of the knee, and the number of TKAs performed is increasing annually worldwide [1]. However, it has been reported that 20–40% of patients undergoing TKA require postoperative blood transfusion therapy [2]. Blood loss not only increases the risks of postoperative infection [3], knee swelling, deep vein thrombosis, transmission of disease, and mortality but also prolongs hospitalisation, affects postoperative functional recovery, and increases medical expenditures [4, 5].

Reducing perioperative bleeding and transfusion rates in patients undergoing TKA is a key measure to accelerate postoperative recovery, shorten hospital stays and reduce health care costs. The application of intraoperative tourniquets and tranexamic acid (TXA) [6] is now a common measure for perioperative blood management [7]. Tourniquets can significantly reduce intraoperative bleeding, maintain clear intraoperative visibility of the surgical field, facilitate the surgical operation and integration of the prosthetic bone interface, shorten the operative time, and reduce postoperative pain and limb swelling, but tourniquets blocking blood flow intraoperatively can increase the incidence of postoperative fibrinolysis and lead to increased bleeding [2].

TXA, one of the commonly used antifibrinolytic drugs in clinical practice, is a lysine synthetic derivative that reversibly blocks the lysine binding site on fibrinolytic enzymes and fibrinogen, resulting in the inability of fibrinogen to bind to fibrin molecules, thus achieving antifibrinolytic effects [8] and antagonising the fibrinolytic hyperactivity stimulated by tourniquet application in terms of mechanism of action to achieve a balance between bleeding and haemostasis [9]. The main methods of administration are intravenous and topical via the joint, and the combination of the two modalities is more effective than either method alone [10–12].

Some scholars currently apply the first dose of intravenous TXA 5 to 30 min prior to skin incision, but a tourniquet in TKA blocks blood circulation to the lower extremity while also affecting blood levels in the operative area [6, 13, 14]. Some authors have intravenously dripped the first dose of TXA before the tourniquet is released [15–17]. There have been no studies comparing the clinical efficacy of these two methods. Therefore, this double-blind randomised controlled study aims to compare the effects of TXA administered intravenously before tourniquet compression and release in primary TKA on the amount of perioperative blood loss and therapeutic efficacy of patients to provide a basis for clinical work.

## Methods

### Patients and design

This prospective, double-blind randomised study was approved by The Institutional Review Board of Panzhihua Central Hospital (no. pzhszxyyll-2022-10) and registered in the Chinese Clinical Trial Registry (ChiCRT-2100053885). Informed consent was obtained before participation in the study. Ninety patients with osteoarthritis of the knee who underwent primary TKA from 01/2022 to 07/2022 in our hospital were selected. All patients 18 years of age or older who were scheduled for primary unilateral TKA for end-stage osteoarthritis were eligible for inclusion. Exclusion criteria: (1) preoperative anaemia or other haematological disorders; (2) preoperative history of severe cardiovascular disease and/or still taking oral anticoagulants; (3) preoperative unilateral lower limb arterial occlusion, venous thrombosis or history of disease; (4) preoperative preexisting autologous blood transfusion or request for blood return device; (5) contraindication for the use of TXA or low molecular weight heparin; (6) perioperative use of erythropoietin, iron, antiplatelet therapy or blood transfusion; (7) preoperative diagnosis of liver disease; (8) BMI > 35; (9) flexion deformity of ≥ 30°, varus-valgus deformity of ≥ 30° (10) inability to cooperate with the trial.

### Treatment groups

In total, 90 patients were randomised to 1 of 2 groups. Group A received intravenous TXA 10 min before tourniquet compression (20 mg/kg) and 3, 6 and 24 h later (10 mg/kg); Group B was treated the same as Group A but received intravenous TXA before tourniquet release. The included patients were randomised into Groups A and B in a 1:1 ratio using the random number table method. The random numbers generated were sealed in opaque envelopes and kept by staff who were not involved in the study and did not measure outcomes. Preoperative, intraoperative and postoperative data were collected and recorded by one researcher without the surgeon being involved in the collection. Throughout the study, the surgeon, patient, and data collector and analyst were unaware of the intervention.

### Surgical procedure

All of the operations were performed by the same team of senior surgeons, all under general anaesthesia. We performed all operations through a midline skin incision and medial parapatellar approach. Intramedullary guides were used for all femoral preparations, and extramedullary guides were used for the tibial preparation. A tourniquet was used throughout the operation to stop bleeding, with a tourniquet pressure of (systolic + 100) mmHg (1 mmHg = 0.133 kPa). A surgeon-selected cemented posterior-stabilised prosthesis (The Legion system (Smith & Nephew, London, UK) was used. The tourniquet was loosened, the bleeding had completely stopped, the wound was flushed with saline and TXA 3 g (30 ml) was injected into the joint cavity, followed by a 60 ml cocktail of 200 mg Ropivacaine (10 ml, 100 mg, Naropin; AstraZeneca AB, Sodertalje, Sweden) and 0.1 ml Betamethasone (1 ml, Diprospan; Merck Sharp & Dohme, Luzern, Switzerland) to reduce postoperative pain prior to capsule closing [18]. Drains was not used postoperatively, and the incision was closed layer by layer and compressed with an elastic bandage [19, 20]. The surgical and tourniquet times and intraoperative blood loss volume were recorded.

### Postoperative management

All patients were managed using the same Postoperative protocols. One gram of TXA (5 ml, 0.5 g; Salvage Pharmaceutical CO.,LTD., Guizhou, China) was given intravenously at 3 and 6 h and on day 2 postoperatively; 1.5 g of Cefuroxime Sodium (1.5 g, Axetine; Medochemie Ltd., Limassol, Cyprus)was given as prophylaxis once every 8 h for a day), Perioperative oral analgesia [21, 22] with 200 mg Celecoxib (200 mg,Celebrex; Pfizer Pharmaceuticals LLC, Vega Baja, Puerto Rico) was given twice a day for 2 weeks postoperatively. An inflatable lower-limb pump was introduced intermittently on the first postoperative day with lower-limb strengthening and physiotherapy. The patients received a half dose of enoxaparin (0.2 ml, 2000 IU, Clexane; Sanofi-Aventis, Gentilly, France) subcutaneously eight hours postoperatively and 4000 IU once a day during hospitalisation, followed by oral rivaroxaban (10 mg, Xarelto; Bayer, Leverkusen, Germany) once a day for ten days after discharge if no bleeding events occurred. Doppler ultrasound examination of both lower limbs was performed in all patients to determine the presence or absence of deep venous thrombosis at discharge and at three weeks and three months after the operation. CT, in combination with clinical symptom assessment, was used to evaluate potential pulmonary disease. All patients were managed through an early rehabilitation program that included muscle power training, passive and active range of motion (ROM) training, and walking training on the second day after the surgery. Blood tests were repeated 1 d, 3 d, 5 d and 21 d postoperatively, and routine follow-up visits at the hospital were performed postoperatively.

### Outcome measurements

Intraoperatively, the amount of bleeding, need for blood transfusion, duration of the operation and duration of tourniquet use were recorded, and the total blood volume was calculated according to the Nadler equation [23]. PBV = k_1_ × height (m) + k_2_ × weight (kg) + k_3_; k_1_ = 0:3669, k_2_ = 0:03219, and k_3_ = 0:6041 for men; k_1_ = 0:3561, k_2_ = 0:03308, and k_3_ = 0:1833 for women. Total blood loss was calculated using the Gross equation [24]: TBL = PBV × (Hct_pre_$-$ Hct_post_)/Hct_ave_, where TBL is the total blood loss, PBV is the patient’s blood volume, Hct_pre_ is the preoperative haematocrit, Hct_post_ is the lowest postoperative haematocrit during hospitalisation or prior to transfusion, and Hct_ave_ is the mean of Hct_pre_ and Hct_post_. Hidden blood loss (HBL) was measured as TBL minus intraoperative blood loss (IBL) [25]. The occurrence of subcutaneous ecchymosis and the circumference of the operated limb were recorded for 6 d after surgery. The rate of knee circumference changes = (knee circumference_post_-knee circumference_pre_)/knee circumference_pre_. The positive, negative and magnitude of the rate of change values indicate the degree of joint swelling or atrophy. Visual analogue scale (VAS) and hospital for special surgery (HSS) scores were collected on the day before surgery and postoperative day1, day 3, day 21 and 3 month, and the postoperative length of stay (LOS), complications and satisfaction were recorded.

### Statistical analysis

The sample size was calculated on the basis of the difference in the primary outcome, namely, total blood loss. Based on pilot data from 24 patients who received unilateral primary TKA with TXA was given intravenously before tourniquet compression between December 2020 and September 2021, the mean total blood loss was 421 ml (SD 272). In the study of TXA was given intravenously before tourniquet release, the mean total blood loss was 642 ml (SD 242) [26]. Based on these parameters, it was determined that 37 patients were required per group, with an assumed alpha of 5% and a power of 95%. To allow for loss to follow-up of 20%, 45 patients were needed for each group. Calculations were performed with G*Power software (Version 3.1.9.7 (Franz Faul, Universitat Kiel, Germany). Statistical analysis was conducted using SPSS for Windows (version 26.0 IBM Corp., Armonk, NY, USA). The categorical variables were analysed using the Pearson chi-square test or Fisher’s precision probability test (sex, side, Kellgren-Lawrence class, transfusion rate, subcutaneous ecchymosis rate, complication rate and satisfaction), whereas the continuous variables were evaluated using Student’s t test (age, weight, height, BMI, preoperative blood volume and laboratory values, operation and tourniquet times, postoperative LOS, blood loss). The variables subjected to multiple comparisons between groups, including decreases in postoperative haemoglobin, haematocrit, red blood cell count, blood loss, HSS and VAS scores, and knee circumference, were analysed using repeated-measures analysis of variance (ANOVA), followed by the Bonferroni-corrected post hoc test. When significant differences were found via repeated-measures ANOVA, Student’s t test was used to determine intergroup significance.

## Results

### Baseline characteristics

**Fig. 1:**
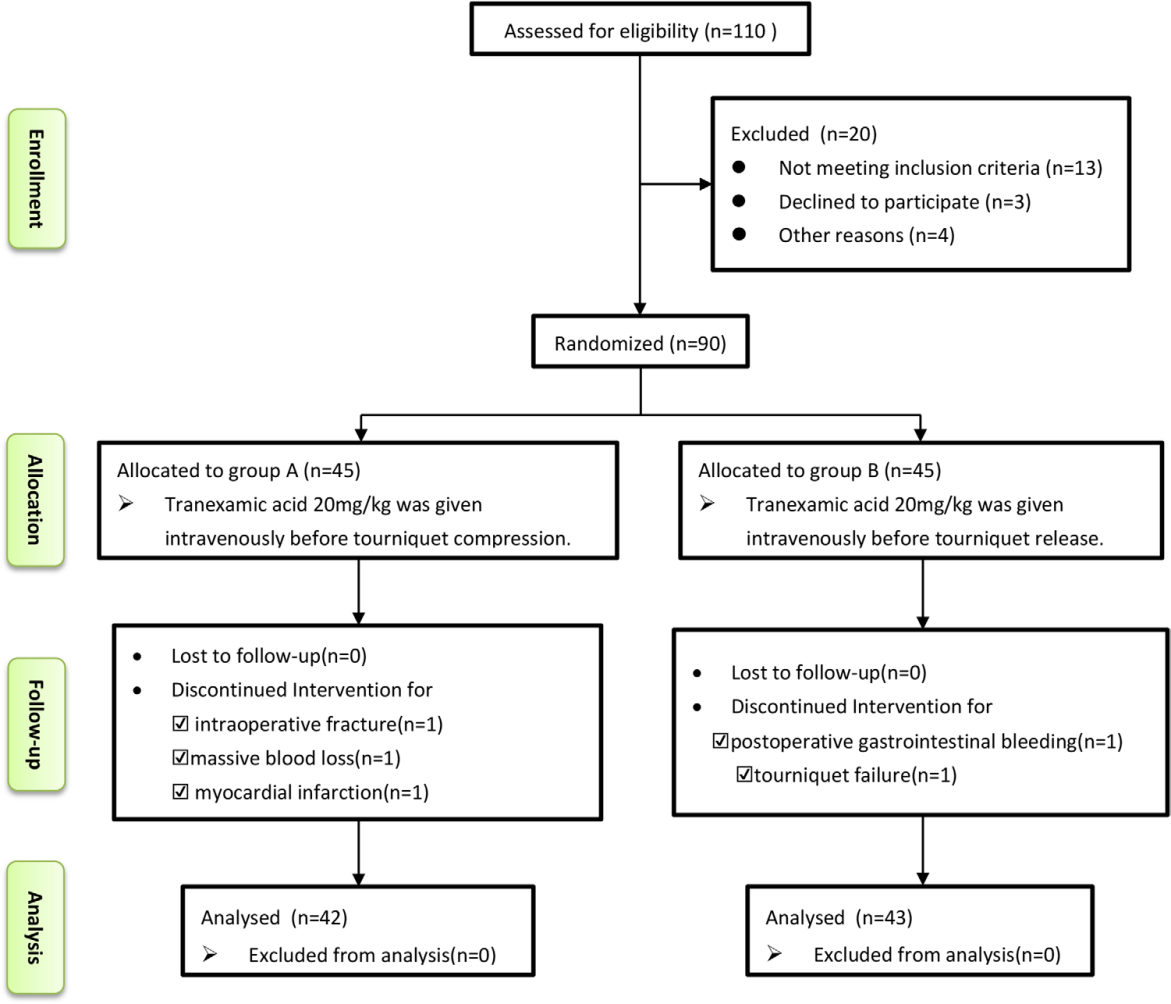
A flow diagram of the study based on the consolidated standards of reporting trials (CONSORT) guidelines

From January 2022 to July 2022, 110 patients scheduled for primary unilateral TKA were screened for participation in our trial. Twenty patients were excluded, and the remaining 90 patients underwent randomisation into Groups A and B. Three patients in Group A were excluded because of intraoperative fracture (1 patient), massive blood loss (1 patient) and myocardial infarction (1 patient). Two patients in Group B were excluded because of postoperative gastrointestinal bleeding (1 patient) and tourniquet failure (1 patient) (Fig. 1). No patient was lost or excluded during follow-up. No significant differences between the 2 groups were identified with respect to demographic data, Kellgren-Lawrence class, preoperative blood volume, operation and tourniquet times, transfusion rate, preoperative HSS score or laboratory values (Table 1).

**Table 1:** Baseline characteristics and demographic characteristics of the 2 groups

Variables	Group A(n = 42)	Group B(n = 43)	*χ*^2^/*t*	*P* Value
Patient characteristics				
Age(year)^b^	67.9 ± 7.6	64.1 ± 9.0	1.966	0.053
Sex(F/M, female%)^a^	32/10(79.4%)	36/7(83.7%)	0.753	0.386
Height(cm)^b^	157.6 ± 6.5	157.5 ± 8.2	0.044	0.965
Weight(kg)^b^	60.3 ± 10.1	61.9 ± 9.1	-0.714	0.478
Body mass index(kg/m^2^)^b^	24.3 ± 4.1	25.0 ± 3.8	-0.791	0.431
Operated side(R/L)^a^	18/24	25/18	1.985	0.159
Kellgren-Lawrence class				
Type III^a^	11	12	0.032	0.859
Type IV^a^	31	31		
Preoperative blood volume(L)^b^	3.7 ± 0.5	3.9 ± 0.4	-0.313	0.755
Preoperative laboratory values				
Haemoglobin (g/L)^b^	131.6 ± 9.9	131.3 ± 12.6	0.111	0.912
Haematocrit (%)^b^	40.5 ± 3.1	40.4 ± 3.7	0.171	0.864
Platelet count(×10^9^/L)^b^	195.4 ± 58.2	201.3 ± 61.6	2.254	0.485
Albumin(g/L)^b^	39.5 ± 2.4	41.3 ± 3.1	1.398	0.782
aPTT(s)^b^	28.0 ± 3.5	27.1 ± 2.5	1.311	57.057
Surgical data				
Operation time(min)^b^	82.5 ± 9.2	84.0 ± 13.3	-0.676	0.501
Tourniquet time(min)^b^	54.7 ± 8.2	57.8 ± 10.4	-1.415	0.161
Transfusion rate(%)^a^	0	0	-	-
LOS postoperatively(d)	7.4 ± 1.9	7.1 ± 1.7	0.648	0.519
Preoperative knee function				
Knee circumference (cm)^b^	36.4 ± 3.0	37.5 ± 2.8	-1.635	0.106
HSS score^b^	51.2 ± 7.3	51.0 ± 9.4	0.091	0.928
VAS score^b^	5.1 ± 1.4	4.9 ± 1.5	0.394	0.695
ROM(°)^b^	105.1 ± 7.1	106.4 ± 8.7	1.428	0.157

*HSS* hospital for special surgery, *VAS* visual analogue scale, *LOS* length of stay, *ROM* range of motion

^a^The values are given as the number of patients

^b^ The values are presented as the mean and the standard deviation

### Intraoperative and postoperative blood loss, haemoglobin and red blood cell count drop values

**Fig. 2:**
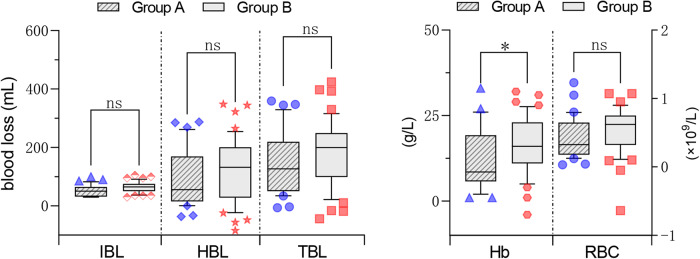
Box plots of blood loss, Hb and RBC postoperatively between two groups. IBL Intraoperative blood loss, HBL Hidden blood loss, TBL Total blood loss, Hb Haemoglobin, RBC Red blood cell, ns No significant difference, *P < 0.05

There was no significant difference in IBL (52.7 ml vs. 63.4 ml, *P* = 0.07), HBL (91.4 ml vs. 119.9, *P* = 0.40) or TBL (144.1 ml vs. 183.3 ml, *P* = 0.72) between Groups A and B. However, there was a tendency for patients in Group B to lose more blood on average than those in Group A; patients in Group A had less of a decrease in haemoglobin than those in Group B (*P* < 0.05). There was no statistically significant difference in the values reflecting the decrease in red blood cell count between the 2 groups, but there was still a tendency for the fluctuations to be more pronounced in Group A (Fig. 2).

### Changes in haemoglobin, haematocrit and red blood cell count (RBC)

**Fig. 3:**
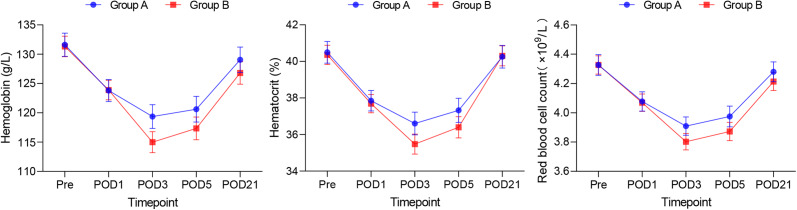
Changes of hemoglobin, hematocrit and RBC in 2 groups

Preoperative haemoglobin, haematocrit and RBC were in the normal range in both groups (Table 1) and started to decline postoperatively, falling to their lowest values 3 days postoperatively before starting to rise again and returning to normal levels by 21 days. There was no significant difference in the comparison of the test values at each follow-up time point. The difference between the 2 groups showed a maximum at postoperative day 3, and the decrease was more pronounced in Group A than in Group B, but the difference was not statistically significant (*P* > 0.05) (Fig. 3).

### Incidence of petechiae and knee circumference changes

**Fig. 4:**
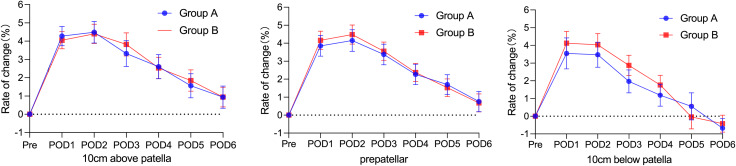
Knee circumference changes of the 2 groups of patients

The incidence of petechiae, mean petechiae area and maximum petechiae area were not significantly different between the 2 groups, with the mean incidence of petechiae remaining at 23.5% and 25.0% and the mean petechiae area being approximately 1.0%, with the maximum petechiae area being 4.0% in Group A and 3.0% in Group B (Table 2). During the perioperative period, the change in the circumference of the limb was the same in both groups, with swelling being more pronounced on days 1–2 postoperatively, subsiding on day 3 and approaching preoperative levels on day 6, but the suprapatellar and prepatellar circumferences were still higher than their preoperative circumferences on day 6, while the infrapatellar circumference was smaller than its preoperative circumference. (Fig. 4).

**Table 2:** Incidence and size of petechiae

Variables	Incidence of petechiae (%)	Average petechiae area (%)	Maximum petechiae area (%)
GroupA(n = 42)	23.5	1.0	4
GroupB(n = 43)	25.0	1.1	3
* χ*^2^/*t*	0.101	-0.571	——
* P* Value	0.75	0.572	——

### Postoperative VAS scores, HSS scores and patient satisfaction

The preoperative VAS scores were 5.1 ± 1.4 and 4.9 ± 1.5 in Groups A and B, respectively, which started to drop postoperatively and gradually decreased to 1.3 ± 0.3 and 1.3 ± 0.6 at 3 months postoperatively. The HSS score decreased from 51.2 ± 7.3 and 51.0 ± 9.4 preoperatively to 48.0 ± 8.3 and 45.7 ± 9.0 on postoperative day 1, began to improve on postoperative day 3 and improved to 81.8 ± 7.5 and 82.4 ± 8.1 at follow-up until 3 months after surgery. However, there was no significant difference in the VAS and HSS scores between the 2 groups at each time point. Patient satisfaction was 97.6% and 95.4% in the 2 groups, respectively (*P* > 0.05).

### Complications

Postoperative complication rates did not differ significantly between the 2 groups (*P* = 0.939), with intermuscular venous thrombosis and wound oozing being more common in both groups. Arteriovenous ultrasound of both lower limbs was repeated 7 days after surgery. Ten patients in Group A and 9 patients in Group B had intermuscular vein thrombosis in the operated limbs (*P* = 0.750). The patients received 4000 IU of enoxaparin subcutaneously every 24 h, and nonweight-bearing functional exercises were performed for 1 week. During postoperative dressing changes, oozing wounds were found in 4 patients in Group A and in 5 patients in Group B (*P* = 0.753). They were treated with wound dressing changes and appropriate pressure dressings and were discharged. None of the patients in either group suffered from lower limb deep vein thrombosis, pulmonary embolism, myocardial infarction, acute renal failure, incisional infection or shock (Table 3).

**Table 3:** Complications

Variables	Group A (n = 42)	Group B(n = 43)	*χ*^2^/*t*	*P* Value
DVT (n)	0	0		
PE (n)	0	0		
Intramuscular venous thrombosis (n)	10	9	0.101	0.750
Cardiac infarction (n)	0	0		
Stroke (n)	0	0		
Acute renal failure (n)	0	0		
Wound complications (n)	4	5	0.099	0.753

*DVT* deep venous thrombosis, *PE* pulmonary embolism

## Discussion

TXA administered intravenously prior to tourniquet compression could effectively reduce blood loss in patients who had undergone total knee arthroplasty. There was no statistical significance in the comparison of intraoperative and postoperative blood loss, postoperative haemoglobin, haematocrit or red blood cell count between the two groups of patients in this study, but the data demonstrated that applying TXA before tourniquet pressurisation had a better haemostatic effect. However, the use of an intraoperative tourniquet and the effect of repelling the blood will have a negative impact on the TXA blood concentration in the tissues of the operated area while blocking the blood circulation of the affected limb [27, 28]. The transient but effective blood concentration of TXA formed in the operative area before tourniquet pressurisation is still effective in blocking fibrinolysis and clot degradation [29–31]. Postoperative subcutaneous petechiae are caused by occult blood loss or subcutaneous capillary haemorrhage induced by anticoagulant drug use, which is an index to support the patients’ postoperative occult blood bleeding, and the results showed that there was no significant difference in the incidence of subcutaneous petechiae or the maximum petechiae area between the two groups of patients. The circumference of the operated limb can reflect the extents of bleeding in the joint cavity and tissue oedema, which affect the effect of postoperative functional exercise and degree of satisfaction during hospitalisation, and routine use of compression stockings can effectively reduce the extent of oedema of the affected limb after surgery, while perioperative swelling of the affected limb in the two groups of patients has a comparable trend of change, with the swelling being more pronounced in the 1–2 days after surgery, beginning to subside in the 3 days after surgery, and approaching the preoperative level in the 6 days after surgery, The result is consistent with Hai-Yan Zhao et al. [32], which is related to the accumulation of fluid in the joint cavity and suprapatellar capsule but also to the use of a postoperative elastic bandage [8].

Multimodal analgesia was strictly applied to both groups, and active and passive functional exercises of the knee joint were strictly performed in the postoperative period. The results showed that there was no difference in the effect of the two regimens on the postoperative pain and function of the patients. During the follow-up period, the pain of the affected limbs was reduced compared with that in the preoperative period, and the improvement in pain was obvious over time; however, due to the influence of postoperative knee swelling, pain and other comprehensive factors, the knee function score decreased first and then increased later, and the improvement in knee function was satisfactory at 3 months after the operation. There was no significant difference in the incidence of complications between the two groups of patients. In this study, some patients had thrombosis of the intermuscular vein of the calf, but there were no obvious clinical symptoms, and the thrombus disappeared after intensive anticoagulation treatment. There was no deep vein thrombosis of the lower extremities or symptomatic pulmonary embolism. Some patients’ wounds showed oozing blood and fluid, which was related to the patients’ postoperative hypoproteinaemia and slow wound healing factors; the state of the wounds improved after dressing changes. None of the patients had complications such as wound infection, nonhealing, acute renal failure, cardiovascular or cerebrovascular accidents, or coagulation abnormalities.

Although this study was well designed, it still has some limitations. First, the study was a single-centre study and the sample size was small. Haemoglobin and blood loss indicators reflected less blood loss and more stable haemoglobin levels in the group of patients who received TXA before loosening the tourniquet, but there was no significant difference in the statistical analysis. Because of the strict inclusion criteria, the sample size may not have been large enough to determine significance for all variables. Second, venography was not used as a standard method to screen for postoperative lower extremity deep vein thrombosis, and Doppler ultrasonography may affect the accuracy of the diagnosis of lower extremity deep vein thrombosis, but the occurrence of lower extremity deep vein thrombosis and symptomatic pulmonary embolism can be determined according to the clinical signs and symptoms at the 1-month postoperative follow-up, and the safety of TXA has been well supported by evidence.

## Conclusion

TXA administered intravenously prior to tourniquet compression could effectively reduce blood loss in patients who had undergone total knee arthroplasty. There were no significant differences in degree of knee swelling, subcutaneous bruising and petechiae, knee function, complication rate, or satisfaction during hospitalisation between the intravenous application of TXA prior to tourniquet compression and the intravenous application of TXA prior to tourniquet loosening in patients who had undergone TKA, but perioperative blood loss was less, as reflected by the changes in both blood loss and haemoglobin with the administration of TXA prior to tourniquet compression.

## Data Availability

The datasets used and analyzed during the current study are available from the corresponding author on reasonable request.

## Abbreviations

IBL: Intraoperative blood loss
HBL: Hidden blood loss
TBL: Total blood loss
Hb: Hemoglobin
RBC: Red blood cell
TKA: Total knee arthroplasty
TXA: Tranexamic acid
VAS: Visual analog scale
HSS: Hospital for Special Surgery
LOS: Length of stay
ROM: Range of motion
DVT: Deep venous thrombosis
PE: Pulmonary embolism
